# Riding the waves: the ongoing impact of COVID-19 on a national surgical training cohort

**DOI:** 10.1007/s11845-021-02739-4

**Published:** 2021-08-27

**Authors:** Orla Hennessy, Amy Lee Fowler, Conor Hennessy, David Brinkman, Aisling Hogan, Emmeline Nugent, Myles Joyce

**Affiliations:** 1 Department of General Surgery, University Hospital Galway, Galway, Ireland; 2 University of Oxford Medical School, England, UK; 3 Royal College of Surgeons in Ireland, Dublin, Ireland

**Keywords:** COVID-19, Reduction, Surgical, Trainee, Training

## Abstract

**Background:**

The World Health Organisation declared a global pandemic on the 11 March 2020 resulting in implementation of methods to contain viral spread, including curtailment of all elective and non-emergent interventions. Many institutions have experienced changes in rostering practices and redeployment of trainees to non-surgical services. Examinations, study days, courses, and conferences have been cancelled. These changes have the potential to significantly impact the education and training of surgical trainees.

**Aim:**

To investigate the impact of the COVID-19 pandemic on training, educational, and operative experiences of Irish surgical trainees.

**Methods:**

Surgical trainees were surveyed anonymously regarding changes in working and educational practices since the declaration of the COVID-19 pandemic on 11 March 2020. The survey was circulated in May 2020 to both core and higher RCSI surgical trainees, when restrictions were at level five. Questions included previous and current access to operative sessions as well as operative cases, previous and current educational activities, access to senior-led training, and access to simulation-/practical-based training methods. A repeat survey was carried out in October 2020 when restrictions were at level two.

**Results:**

Overall, primary and secondary survey response rates were 29% (*n* = 98/340) and 19.1% (*n* = 65/340), respectively. At the time of circulation of the second survey, the number of operative sessions attended and cases performed had significantly improved to numbers experienced pre-pandemic (*p* < 0.0001). Exposure to formal teaching and education sessions returned to pre-COVID levels (*p* < 0.0001). Initially, 23% of trainees had an examination cancelled; 53% of these trainees have subsequently sat these examinations. Of note 27.7% had courses cancelled, and 97% of these had not been rescheduled.

**Conclusion:**

Surgical training and education have been significantly impacted in light of COVID-19. This is likely to continue to fluctuate in line with subsequent waves. Significant efforts have to be made to enable trainees to meet educational and operative targets.

## Introduction

The novel coronavirus, SARS-CoV-2 (COVID-19), was declared a pandemic on the 11 of March 2020 by the World Health Organisation (WHO) [1]. At that time, Europe was becoming the new epicentre of the disease [2]. In response to this, many EU nations, including the Republic of Ireland, introduced stringent measures in an attempt to limit the spread of disease. These measures included significant travel restrictions as well as closure of many non-essential services.

Within the healthcare setting, working practices changed dramatically. All non-emergent and non-time sensitive elective activities were cancelled. Within each institution, rosters were altered to limit staff exposure, and in some cases, surgical staff were redeployed to areas of projected need including medical and anaesthetic teams. Additionally, many institutions saw a reduction in regular teaching activities such as journal clubs and grand rounds. At a national level, all surgical training days, exams, and courses were officially cancelled or postponed by the Royal College of Surgeons in Ireland (RCSI). As the pandemic has progressed, services have experienced varying levels of restrictions in line with each “wave” of COVID-19 infections and national government recommendations.

These ongoing changes have contributed to altered working hours, educational activities, and operative exposure for surgical trainees across all specialties and levels. As we cycle through varying levels of national lockdown, our aim is to investigate the ongoing impact of these changes and gather the opinions of trainees at a national level in order to guide education and training throughout subsequent waves.

## Methods

Data were collected by means of an online survey, which was distributed over a 4-week period from 2 to 30 May 2020. This period was during the “1st wave” of COVID-19 in Ireland, during which restrictions were at level five. A second survey was distributed over a 2-week period between 14 and 28 of October 2020. During this period, restrictions had been eased to level two. The survey was completed via Google™ forms and was designed by the authors, *OH*, *ALF*, *MJ*, *AH*, *and EN,* with group consensus on questions to be included. The survey was circulated to RCSI core (CST) and higher (HST) surgical trainees electronically via the RCSI postgraduate mailing list and was promoted via cohort-specific WhatsApp and social media groups. Participants were surveyed voluntarily and anonymously regarding changes in working and educational practices since the declaration of the COVID-19 pandemic on 11 March 2020. Completion of the survey was deemed to infer consent. Participants were deemed eligible if they were currently enrolled in the RCSI core or higher surgical training programmes.

The survey was explained to participants with a brief introductory section and was comprised of five main sections, with a combination of multiple choice and free text responses. "Demographic data" collected basic demographic and training information. "Changes to working hours and redeployment" assessed changes in working hours, call rosters, and redeployment. "Changes to operative practices" compared previous to current operative experience. "Changes in educational and training activities" compared previous to current educational activities, access to senior-led training, and access to simulation-/practical-based training methods. The final section was composed of free text answer questions in which trainees could express additional concerns and suggestions.

Responses were collected securely after the closing date, via a downloaded CSV (comma-separated values) file and stored in a password-protected Excel file (version 16.38) on an encrypted computer.

### Statistical analysis

Data were graphed and analysed in GraphPad Prism (version 8.0.0 for Windows, GraphPad Software, San Diego, California USA). Data were assessed for normality using the D'Agostino and Pearson omnibus normality test. The normality test was failed, and as such, a Kruskal–Wallis test was used to compare the means across the three groups. The post-hoc test used was Dunns multiple comparisons test. Data were graphed as mean + / − SEM, with *p* > 0.05 considered significant. Significance was represented as follows: *p > 0.05, **p > 0.01, ***p > 0.001, and ****p > 0.0001.

## Results

### Demographic data

For round 1, 29% (*n* = 98/340) of current surgical trainees in the Republic of Ireland responded. Of these, 44% (*n* = 43) were from the core surgical training (CST) pathway, and 46% (*n* = 45) were from the higher surgical training (HST) pathway. For round 2, response rate was 19% (*n* = 65/340) with 25% of respondents (*n* = 16) on the core and 75% of respondents (*n* = 49) on the higher surgical training pathways, respectively. Breakdown of respondents demographics by specialty and level of training is outlined in Table 1.

**Table 1:** Trainee demographics by training year and specialty

**Variable**	**Round 1**	**%**	**Round 2**	**%**
**Current training year**				
ST1	26	26.50%	5	7.7%
ST2	17	17.30%	11	16.9%
ST3	17	17.30%	11	16.9%
ST4	9	9.20%	10	15.4%
ST5	11	11.20%	4	6.2%
ST6	7	7.10%	8	12.3%
ST7	5	5.10%	6	9.2%
ST8	4	4.10%	10	15.4%
ST8 +	2	2%	0	0%
**Current specialty HST**				
General	21	32.30%	18	33.3%
Vascular	8	12.30%	4	7.4%
Orthopaedics	21	32.30%	7	13%
Urology	7	10.80%	4	7.4%
Cardiothoracics	4	6.20%	1	1.9%
ENT	2	3.10%	6	11.1%
Opthalmology	1	1.50%	0	0%
Paediatrics	1	1.50%	2	3.7%
Plastic surgery	0	0%	12	22.2%
**Current Specialty CST**				
General	21	46.70%	11	44%
Vascular	7	15.60%	1	4%
Orthopaedics	9	20%	5	20%
Urology	2	4.40%	2	8%
Cardiothoracics	2	4.40%	0	0%
ENT	1	2.20%	1	4%
Opthealmology	1	2.20%	0	0%
Paediatrics	0	0%	3	12%
Plastic surgery	2	4.40%	2	8%

### Changes to working hours and redeployment

**Fig. 1:**
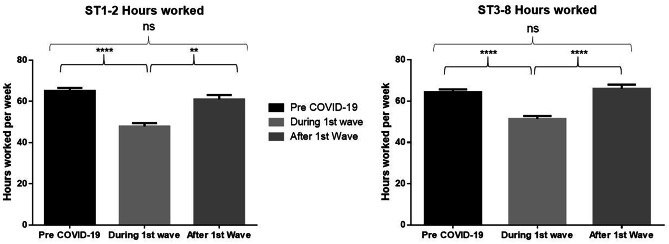
**A**n initial significant reduction followed by restoration of working hours at both CST and HST level. During the first wave refers to data from the May survey, with after the first wave referring to the October survey

Overall, trainees experienced a significant reduction in working hours (64.4 + / − 9.5 vs 49.4 + / − 9.8 h, *p* < 0.0001) during the COVID-19 pandemic. This was seen to be significant at both CST (65.1 + / − 9.8 h pre-COVID versus 47.9 + / − 10.1 h post-COVID, *p* < 0.0001) and HST level (64.4 + / − 9.7 h pre-COVID versus 51.4 + / − 10.2 h post-COVID, *p* < 0.0001). In the second round, working hours had returned to pre-lockdown levels, with no significant difference between time points. This is seen at both CST (*p* < 0.0001) and HST (*p* < 0.0001) level as illustrated in Fig. 1.

Almost 10% (*n* = 9) of respondents were redeployed outside of surgical services to areas such as ED (*n* = 4), ICU (*n* = 3), COVID teams (*n* = 1), and medical teams (*n* = 1). All of those redeployed (*n* = 9) were at CST level. At the time of the second survey, no trainees reported being redeployed.

### Changes to operative practices

During the first survey period, trainees at both CST and HST level experienced a significant reduction in access to elective major sessions (CST *p* < 0.0001, HST *p* < 0.0001) day-case (CST *p* < 0.0001, HST *p* < 0.0001) and endoscopic sessions (CST *p* < 0.001, HST *p* < 0.0001). Interestingly, CST trainees experienced a significant decrease in emergency sessions (*p* < 0.0001); however, HST trainees were not significantly affected (*p* > 0.05). In terms of supervised operating, trainees at all levels were performing less elective major (CST *p* < 0.0001, HST *p* < 0.0001), day-case (CST *p* < 0.0001, HST *p* < 0.0001), endoscopy (CST *p* < 0.05, HST *p* < 0.0001), and emergency procedures (CST *p* < 0.0001, HST *p* < 0.0001).

**Fig. 2:**
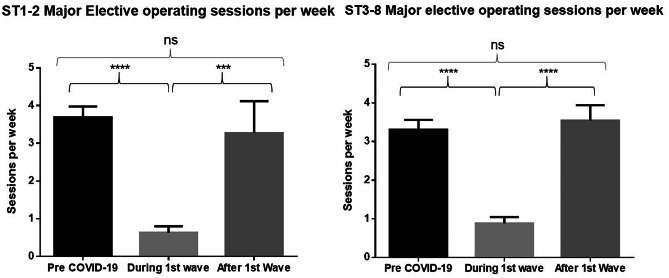
An initial significant reduction followed by restoration of major elective sessions at both CST and HST level. During the first wave refers to data from the May survey, with after the first wave referring to the October survey

**Fig. 3:**
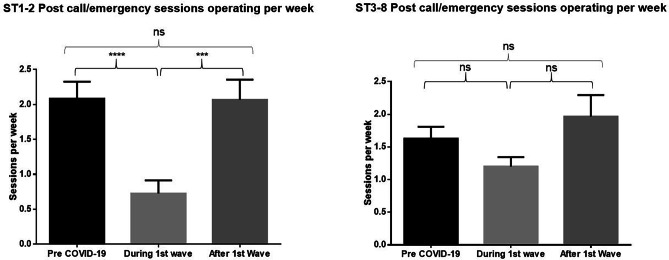
An initial significant reduction followed by restoration of emergency sessions at both CST and HST level. During the first wave refers to data from the May survey, with after the first wave referring to the October survey

Major elective and emergency session numbers returned to pre-COVID levels in the second round, with no significant difference found at CST or HST level. This is illustrated clearly in Figs. 2 and 3. Additional figures for other operative sessions as well as full statistical analysis are available on request.

### Changes in educational and training activities

**Fig. 4:**
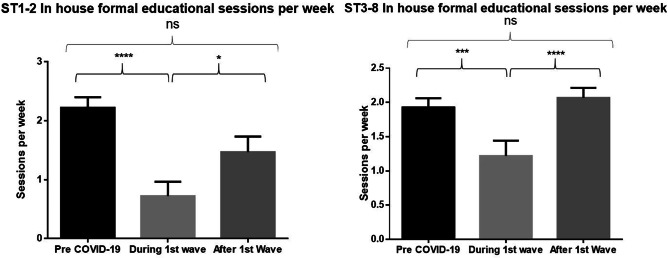
**A**n initial significant reduction followed by restoration of local formal educational sessions at both CST and HST level

Overall, trainees saw a significant drop in formal educational sessions per week (mean pre-COVID 2.0 SD + / − 1.0, mean post-COVID 0.9 + / − 1.4 *p* < 0.0001). 55.4% (*n* = 55) of trainees reported that this was in virtual format. 76.5% (*n* = 75) of trainees have had additional virtual sessions organised by their training body or college. At the second time point, local teaching had returned to pre-COVID levels with no significant difference between the time points at CST or HST level as illustrated in Fig. 4.

In terms of other educational activities, 20.4% (*n* = 20) of trainees had a mandatory course cancelled. As of the second time point, 97% of mandatory courses remained cancelled and had yet to be rescheduled. Furthermore, 18.3% (*n* = 18) of trainees had a mandatory examination cancelled. By the second time point in October, 53% of those with cancelled examinations had subsequently been rescheduled. Disappointingly, only 35.7% (*n* = 35) of trainees were aware of the presence of simulation facilities within their hospital.

### Comments from trainees

In the final section of the survey, trainees were given a chance to provide commentary, suggestions, and express concerns. Trainees felt that COVID-19 had impacted significantly on access to elective (83.7%, *n* = 82) and emergency cases (73.5%, *n* = 72). Trainees expressed concern regarding this lack of access to theatre lists and the potential long-term impact on operative experience. Some trainees expressed feeling unprepared to progress from CST to HST level (*n* = 3). In the second round, 66% (*n* = 43) trainees expressed that they felt that trainees should have to ability to follow and were agreeable to following consultant trainers in instances where public lists were being moved to private facilities as part of the National Treatment Purchase Fund (NTPF) or in response to COVID surges.

When asked to suggest alternative methods for delivering both practical and educational sessions, the majority of comments suggested the use of virtual teaching (*n* = 47 comments, 94%) and simulation (*n* = 17 comments, 35.4% of responses) for technical skills.

## Discussion

COVID-19 has changed the face of surgical practice worldwide and has drastically affected access to training worldwide. Elective and emergency operating has decreased significantly in light of perceived increased risk to patients and healthcare workers. Early correspondence from surgical groups globally suggested the need for reduced exposure of surgical staff and patients to operative risk, recommending a higher threshold for operative intervention and reduced involvement of non-essential team members, specifically trainees, in the operating theatre [3, 4].

Decreased numbers of operative cases combined with redeployment, changes to working hours, and reduction in formal surgical training have had huge consequences for surgical training. Our study demonstrates the negative impact of COVID-19 on surgical training within the Republic of Ireland across specialties and levels of training during stringent lockdowns. Access to elective operating reduced across CST and HST levels. Whilst HST trainees were still getting access to emergency cases, volume of caseload performed with a trainer or assisted was significantly less (cases performed: 2.6 ± 2.1 vs. 1.6 ± 1.7, *p* < 0.01; cases assisted: 1.8 ± 1.4, vs. 1 ± 0.1, *p* < 0.001). This is reflected in many other studies worldwide [5–11]. The COVID-STAR Collaborative released an online survey assessing UK-based trainees across all deaneries and training grades between 11 May and 8 June 2020. They received 810 responses and reported a significant loss of access to elective (69.5%), endoscopic (69.5%), and emergency (48.5%) training and, overall, noted a significant impact on surgical training irrespective of training level or specialty [10].

As experienced so far in the course of the pandemic, restrictions can be expected to change in tandem with infection numbers, resulting in ongoing disruption to surgical training. As illustrated through our survey, opportunities for training are intimately linked to COVID-19 case numbers and national recommendations. It can be expected that trainee case numbers will continue to rise and fall accordingly. Fortunately, as seen in our second survey, training numbers do seem to rapidly return to pre-lockdown levels with resumption of services. What remains to be seen is the number of waves of lockdown to come. Hopefully, with vaccination programmes rolled out within our hospitals and national rollouts ongoing, there is light at the end of the tunnel.

Interestingly in our second survey, whilst operative numbers returned to normal levels, disruptions to the delivery of mandatory courses and training events persisted. A striking figure is that over 97% of cancelled courses, a proportion of which are mandatory for training and progression, had yet to be rescheduled. Thus, the focus must shift towards development of technologies and techniques to aid surgical education. The pandemic brings an opportunity for surgical learners and their trainers to restructure and adapt surgical training strategies. The lack of in-person didactic teaching can be mitigated by access to e-learning material, virtual lectures via platforms such as Zoom (Zoom Video Communications, Inc., San Jose, CA, USA) or Microsoft Teams (Microsoft Corporation, Redmond, Washington) and access to independent learning resources [11]. Nationally, 75% of trainees surveyed have access to virtual teaching sessions arranged by their training programme, which has been well received anecdotally. This is a resource which has been invaluable and should be considered as a long-term option for surgical training, with assigned protected training time. Worldwide, the surgical community is adapting to similar platforms to deliver conferences, with some moving to a virtual or hybrid format and increasing access to web-based content [12–16]. RCSI has also taken many steps in increasing access to training and educational material. During COVID-19, a weekly webinar series was launched, which focuses on different topics and covers all surgical specialties. Overall, this has been well received. The annual bootcamp for new surgical trainees was carried out in a blended fashion incorporating small groups as well as a virtual platform, and again the feedback has been positive.

The training of operative skills in the setting of reduced theatre time becomes a more difficult problem to tackle. One strategy presented by Coe et al. is operative video-based education (VBE). This has been shown to be an effective method for acquisition of knowledge and operative preparation [11, 17]. Web and phone-based platforms which are accessible to trainees at home to help mitigate some of the diminished operative learning [18]. Prior to the outbreak of COVID-19, there was already an established utility for simulation in surgical training [19–23]. Simulation may be particularly helpful for training practical motor skills in more junior trainees [19, 22, 24], who have been demonstrated to be more affected by lack of operative exposure. This may also allow trainees to optimise and make more efficient use of limited theatre time [23, 25, 26]. With the wealth of innovation available to us, efforts should focus on promoting and developing these platforms and ensuring access for trainees [27].

The continued impact of COVID-19 on training must be mitigated to prevent further impact on trainees. At a local level, every surgical training opportunity must be utilised across all settings including multi-disciplinary meetings, outpatient activities and ward rounds [28]. At a hospital level, it is crucial to ensure sites exist where operating can continue irrespective of COVID-19 status and restrictions. Experiences of operating and training in the private sector have been mixed dependent on region and specialty. RCSI and specialty associations must ensure trainee access to these cases and must promote equal access across regions. RCSI has invested significant research into simulation technologies in recent years, and these technologies should be used to counter any shortfall in operative levels. The protection of access to simulation-based training within the RCSI and access to local surgical skills areas should be provided to all training centres. The RCSI and relevant training bodies must continue to support surgical recovery and ensure that training is at the forefront of these discussions. The RCSI must work with specialty associations to ensure that all trainees have equal access and opportunity to all training and teaching, irrespective of specialty and grade. Trainers and educational supervisors must ensure they address individual trainee concerns and shortfalls and ensure an action plan early in the training year, to be reviewed frequently. Specialty bodies must ensure that flexibility is provided to trainees to ensure discrepancies in training can be recovered.

### Study limitations

Whilst our initial survey response rate was fair, with a response rate of 29%, the authors are aware that some groups may have minimal representation in our findings. Lack of engagement in these groups may be due to survey fatigue or having a particularly positive or negative experience through the COVID-19 emergency. This is reflected in the poorer response to the second survey of 19.1%. Whilst this may lead to an element of reporting bias, our experience is similar to that collected in other training cohorts internationally [10]. Data will be made available upon request to all trainees and associated training bodies.

#### Conclusion

The COVID-19 pandemic has resulted in a paradigm shift in delivery of surgical services and by, proxy, in surgical training. Our survey results are analogous to similar studies in other countries, indicating a significant impact on operative, educational, and training output in the Republic of Ireland, and our use of two separate time points illustrates that training is likely to continue to rise and fall in tandem with national restrictions. Endeavours must now focus on ensuring access to training opportunities and protected training time, as well as integration of virtual and simulation teaching platforms to the training repertoire. Most importantly, we must work in tandem as trainees and trainers to produce an efficacious surgical training programme which we can take forward in the wake of COVID-19.

## Data Availability

All data is fully anonymised and freely available through the corresponding author upon request.
